# Clinical characteristics and survival outcomes in patients with pulmonary sarcomatoid carcinoma: a multicenter retrospective study

**DOI:** 10.1007/s12094-024-03823-8

**Published:** 2024-12-25

**Authors:** Zhijuan Du, Yuhui Qin, Yahui Lv, Jie Gao, Siyuan Chen, Xiangyu Du, Tao Li, Yi Hu, Zhefeng Liu

**Affiliations:** 1 Medical School of Chinese PLA, Beijing, China; 2 Department of Medical Oncology, The First Medical Center of Chinese PLA General Hospital, Beijing, China; 3 Department of Pathology, The First Medical Center of Chinese PLA General Hospital, Beijing, China; 4 State Key Laboratory of Molecular Oncology, CAMS Key Laboratory of Translational Research on Lung Cancer, Department of Medical Oncology, National Cancer Center/National Clinical Research Center for Cancer/Cancer Hospital, Chinese Academy of Medical Sciences and Peking Union Medical College, Beijing, China; 5 Department of Medical Oncology, Senior Department of Oncology, The Fifth Medical Center of Chinese PLA General Hospital, Beijing, China

**Keywords:** Pulmonary sarcomatoid carcinoma, NSCLC, Immunotherapy, Survival outcomes, Retrospective study

## Abstract

**Purpose:**

The clinicopathologic features, mutational status, immunohistochemical markers, and prognosis of Pulmonary sarcomatoid carcinoma (PSC) remain uncertain.

**Methods:**

This study included 81 PSC and 337 lung adenocarcinomas (LUAD). Progression-free survival (PFS), overall survival (OS), and other clinical data were examined.

**Results:**

46% PSC patients harbored KRAS mutation and 23% harbored EGFR mutation. Univariable analysis identified type and cTNM stage as significant predictor of PFS (type: HR 0.216; 95% CI 0.133–0.349; P < 0.001, cTNM stage: HR 0.483; 95% CI 0.269–0.846; P = 0.014) and OS (type: HR 0.269; 95% CI 0.156–0.465; P < 0.001, cTNM stage: HR 0.435; 95% CI 0.219–0.865; P = 0.018). Multivariable analysis confirmed sex, type and cTNM stage as independent predictors of PFS (sex: HR 2.026; 95%CI 1.027–3.996; P = 0.042; type: HR0.140; 95% CI 0.083–0.238; P < 0.001, cTNM stage: HR0.305; 95% CI 0.165–0.564; P < 0.001) and OS (type: HR0.231; 95% CI 0.132–0.404; P < 0.001, cTNM stage: HR 0.394; 95% CI 0.194–0.797; P = 0.010). Significant differences in PFS (P < 0.0001) and OS (P = 0.022) were observed between PSC and LUAD, and for PC compared with SCC (PFS: P = 0.00036, OS: P = 0.0053). Additionally, PSC patients treated with immunotherapy showed significantly better OS (P = 0.0019) compared with those treated without immunotherapy.

**Conclusions:**

PSC exhibits high KRAS and EGFR mutation rates, and spindle cell carcinoma has a worse prognosis. Immunotherapy shows potential as a treatment for advanced PSC.

**Supplementary Information:**

The online version contains supplementary material available at 10.1007/s12094-024-03823-8.

## Introduction

Pulmonary sarcomatoid carcinoma (PSC) is one of the rarest subtypes of non-small cell lung cancer (NSCLC) [1], which was initially reported in the World Health Organization’s (WHO) 1999 lung tumors classification as “carcinoma with sarcomatoid, pleomorphic or sarcomatous elements” [2]. Pleomorphic carcinoma (PC), carcinosarcoma (CS), and pulmonary blastoma (PB) are the three subgroups in the latest 2021 WHO classification. Within PC, there are two subtypes: spindle cell carcinoma (SCC) and giant cell carcinoma (GCC) [3]. Due to the unique pathological features of each subtype, the prognosis varies among the different subtypes [4].

PSC is more frequently seen in males and has a strong link to tobacco exposure [5]. It is marked by high heterogeneity and poor differentiation, which limits the available treatment options for this disease [6]. Results from a multicenter, single-arm, open-label phase II study showed that savolitinib exhibited acceptable safety and promising activity in patients with PSC and other subtypes of NSCLC who had positive METex14 skipping mutations [7]. ICIs have demonstrated encouraging outcomes in the treatment of advanced PSC, and their combination with anti-angiogenic therapy could represent a more potent therapeutic strategy for this disease [8]. Multiple studies have examined the histological and genetic traits of PSC, finding an increased prevalence of mutations in genes like EGFR, KRAS, ALK, and MET, along with observed PD-L1 expression [9, 10]. A study has indicated that MUC4 and GATA3 can serve as reliable immunohistochemical (IHC) markers for distinguishing PSC [11]. Additionally, the expression of immune-related proteins such as PD-L1, CD8, CD4, FOXP3, and LAG3 are strongly linked to the prognosis of PSC patients [12].

As a rare type of NSCLC, PSC was reported to range from 0.5 to 1% among all cases of lung malignancies [6, 13]. To date, reports on PSC with a large sample size are still scarce in the literature [7, 14]. Due to its low incidence, the best treatment approach for this special subtype of NSCLC remains controversial [7, 15]. Therefore, this study retrospectively analyzed PSC cases in three centers, focusing primarily on the clinicopathological and prognostic characteristics, with the aim of enhancing comprehension of this uncommon ailment.

## Materials and methods

### Data collection

This study was a multicenter retrospective analysis of PSC patients, including a comparative analysis of two subgroups. The patient cohort was created using the Chinese PLA General Hospital Case Database and involved 3 academic centers. The inclusion criteria required a PSC diagnosis with histological or cytological examination conducted between January 1, 2010 and September 25, 2023. Patients without relevant clinical information in the medical record were excluded. All patients underwent radiological evaluations, which included chest CT scans, abdominal CT scans or ultrasound, brain CT scans or MRIs, and radionuclide bone scans in order to verify the diagnosis of primary lung tumor and TNM stage (according to the International Association for the Study of Lung Cancer, 8th edition). Experienced pulmonary pathologists in our hospital conducted microscopic evaluations of the IHC stained sections.

A total of 81 PSC patients and 337 LUAD patients were enrolled in the study. Basic demographic and clinical data, such as age, sex, histopathological findings, TNM staging, treatment initiation and completion dates, progression-free survival (PFS), and overall survival (OS), were retrieved from the electronic medical record system. PFS is defined the duration from the start of treatment until disease progression or death, and OS is defined as the time from treatment initiation to death from any cause. Propensity score matching (PSM) strives to balance treatment groups concerning measured baseline covariates, thereby facilitating a comparison with diminished selection bias. This valuable statistical approach emulates the randomized controlled trial (RCT) [16]. PSM was used in this study with a caliper of 0.02. A 1:1 matching was conducted for 53 PSC patients and 53 LUAD patients based on age, sex, smoking history, tumor family history and cTNM stage. After matching, the standardized mean differences (SMDs) for all covariates were less than 0.001, indicating good balance between the treatment groups. Data on disease recurrence and survival were collected though telephone interviews or follow-up appointments at clinics. The last follow-up visit was on November 14, 2023. Data collection procedures complied with the Declaration of Helsinki and received approval from the Institutional Review Board of PLA General Hospital in Beijing, China (approval number: S2021-256-02).

### Statistical analysis

The clinical characteristics of patients and the status of their diseases were outlined through descriptive analyses. The PFS duration was computed from the date of initial PSC diagnosis to disease progression, while the OS duration was measured from the time of initial PSC diagnosis to either death or the last recorded contact for patients still alive. Univariable and multivariable analyses were conducted. The association between PSC subtype and both PFS and OS was assessed using the Cox proportional hazards regression model, estimating the hazard ratio (HR) and significance of each subtype. The threshold for statistical significance in all analyses was set at P < 0.05. All the data above were analyzed using R 4.3.1 or SPSS 25.0.

## Results

### Clinical characteristics

111 patients with PSC were screened and a total of 30 patients were excluded: 16 patients had no baseline and 14 had no follow-up information. Finally, the study included 418 patients, comprising 81 cases of PSC and 337 cases of LUAD. The median follow-up time is 26.7 months, 95% CI: [0.95, NA]. Of the 81 PSC patients diagnosed at 3 academic centers, 28 patients were classified as PC, 20 were SCC, 3 were GCC, 5 were SC, 2 were PB and 23 were not classified (Supplementary Fig. S1). Of the cases above, 86.4% (n = 70) were male and 13.6% (n = 11) were female; median age of patients is 59.6 years (range from 12 to 78). Age distribution did not differ significantly between PSC and LUAD groups; however, the former exhibited a higher proportion of male patients (P = 0.003), longer smoking history (P = 0.003), a higher incidence of tumor family history (P < 0.001), and more advanced stage of the disease (P < 0.001). (Table 1).

**Table 1:** Clinical characteristics of patients with PSC and LUAD

	PSC (n = 81)	LUAD (n = 337)	P value
*Age*			0.484
< 60	41(20.8)	156(79.2)	
≥ 60	40(18.1)	181(81.9)	
*Sex*			**0.003**
Male	70(22.9)	236(77.1)	
Female	11(9.8)	101(90.2)	
*Smoking* *history*			
Ever	59(22.3)	205(77.7)	**0.003**
Never	22(16.7)	132(83.3)	
*Tumor family history*			**< 0.001**
Yes	61(28.4)	154(71.6)	
No	20(9.9)	183(90.1)	
*cTNM stage*			**< 0.001**
I–IIIA	42(65.6)	22(34.4)	
IIIB–IV	39(11.0)	315(89.0)	

Bold values indicate results with statistical significance (P < 0.05)

LUAD, lung adenocarcinomas; PSC: pulmonary sarcomatoid carcinoma

### Mutational status and immunohistochemical staining of PSC

Out of 19 patients tested, mutations were detected in thirteen (68.4%) of them in the genes we examined. 6 of 13 (46%) patients harbored KRAS mutation, 3 of 13 (23%) harbored EGFR mutation and 4 of 13 (31) harbored other mutation. Among these patients, one (case 14, Supplementary Table S1) carried mutations in KRAS (exon 2), ERBB4 (exon 15), and TP53. No fusions involving ALK, RET, or ROS1 were detected (Supplementary Table S1).

**Fig. 1:**
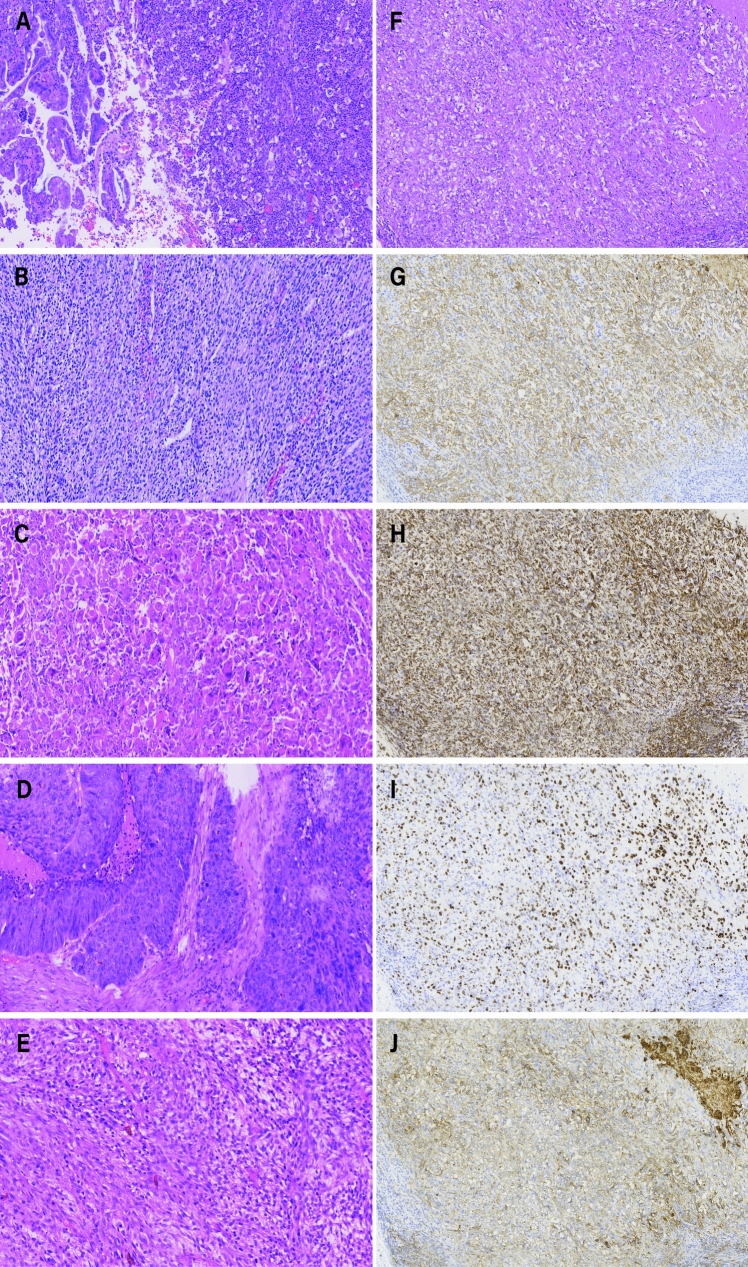
The immunohistochemical (IHC) stained sections. Different subtypes of pulmonary sarcomatoid carcinoma (PSC) exhibit distinct histological characteristics in IHC-stained sections: the adenocarcinoma component with large tumor cells in pleomorphic carcinoma (PC) (**A**); spindle cell carcinoma (SCC) composed solely of spindle cells (**B**); giant cell carcinoma (GCC) showed large tumor cells (**C**); and carcinosarcoma (CS) contains histological elements of both carcinoma (**D**) and sarcoma (**E**). **F–J** are different stains of consecutive sections. The tumor is positive for CK (**G**), Vimentin (**H**), Ki-67 (**I**) and PD-L1 (**J**)

IHC staining results showed that CK (75 of 80, 93.8%), Vimentin (67 of 75, 89.3%), TTF-1 (33 of 80, 41.3), CK7 (40 of 60, 66.7%), P40 (5 of 30, 16.7%), P63 (17 of 56, 30.4%), EMA (6 of 12, 50.0%), PD-L1 (13 of 23, 56.5%), and Ki-67 (69 of 75, 92%) were expressed in different frequency (Supplementary Table S2). The IHC staining results are shown in Fig. 1.

### Survival analysis

Before PSM, univariable analysis revealed that type is significant predictors of PFS (hazard ratio (HR) 2.240; 95% confidence interval (CI) 1.698–2.955; P < 0.001) and type and cTNM stage were significant predictors of OS (type: HR 1.422; 95% CI 1.050–1.925; P = 0.023, cTNM stage: HR 0.467; 95% CI 0.308–0.708; P < 0.001) (Supplementary Table S3). The type and cTNM stage were significant predictors of PFS (type: HR 7.273; 95% CI 5.002–10.535; P < 0.001, cTNM stage: HR 0.181; 95% CI 0.115–0.287; P < 0.001) and OS (type: HR 2.850; 95% CI 2.005–4.052; P < 0.001, cTNM stage: HR 0.260; 95% CI 0.162–0.416; P < 0.001) in multivariable analysis (Supplementary Table S4). After PSM, univariable analysis revealed that type and cTNM stage were significant predictors of PFS (type: HR 0.216; 95% CI 0.133–0.349; P < 0.001, cTNM stage: HR 0.483; 95% CI 0.269–0.864; P = 0.014) and OS (type: HR 0.269; 95% CI 0.156–0.465; P < 0.001, cTNM stage: HR 0.435; 95% CI 0.219–0.865; P = 0.018) (Table 2). The sex, type and cTNM stage were still significant predictors of PFS (sex: HR 2.026; 95%CI 1.027–3.996; P = 0.042; type: HR 0.140; 95% CI 0.083–0.238; P < 0.001, cTNM stage: HR 0.305; 95% CI 0.165–0.564; P < 0.001) and OS (type: HR 0.231; 95% CI 0.132–0.404; P < 0.001, cTNM stage: HR 0.394; 95% CI 0.194–0.797; P = 0.010) in multivariable analysis (Table 3).

**Table 2:** Univariable analyses for PFS and OS in patients with PSC and LUAD after PSM

Variables	PFS			OS			
	HR	95%CI	*P*-value		HR	95%CI	*P*-value
Age	0.778	0.495–1.223	0.277	0.677		0.404–1.137	0.141
Sex (male/female)	1.424	0.731–2.774	0.299	1.413		0.668–2.989	0.366
Type	0.216	0.133–0.349	**< 0.001**	0.269		0.156–0.465	**< 0.001**
cTNM stage	0.483	0.269–0.864	**0.014**	0.435		0.219–0.865	**0.018**

Bold values indicate results with statistical significance (P < 0.05)

HR, hazard ratio; LUAD, lung adenocarcinomas; OS, overall survival; PFS, progress-free survival; PSC, pulmonary sarcomatoid carcinoma; PSM, propensity score matching

**Table 3:** Multivariable analyses for PFS and OS in patients with PSC and LUAD after PSM

Variables	PFS			OS			
	HR	95%CI	*P*-value		HR	95%CI	*P*-value
Age	0.713	0.450–1.131	0.151	0.602		0.356–1.020	0.059
Sex (male/female)	2.026	1.027–3.996	**0.042**	1.588		0.748–3.371	0.229
Type	0.140	0.083–0.238	**< 0.001**	0.231		0.132–0.404	**< 0.001**
cTNM stage	0.305	0.165–0.564	**< 0.001**	0.394		0.194–0.797	**0.010**

Bold values indicate results with statistical significance (P < 0.05)

HR, hazard ratio; LUAD, lung adenocarcinomas; OS, overall survival; PFS, progress-free survival; PSC, pulmonary sarcomatoid carcinoma; PSM, Propensity score matching

**Fig. 2:**
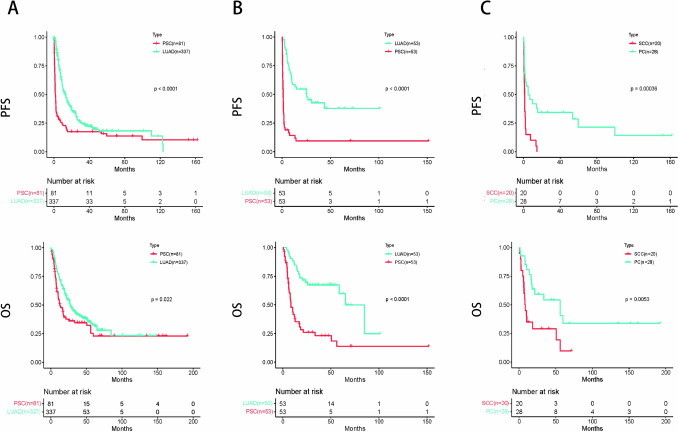
Kaplan–Meier survival curves. Progression-free survival (PFS) and overall survival (OS) according to lung adenocarcinoma (LUAD) and pulmonary sarcomatoid carcinoma (PSC) before PSM (**A**). PFS and OS according to LUAD and PSC after PSM (**B**). PFS and OS according to pleomorphic carcinoma (PC) and spindle cell carcinoma (SCC) (**C**)

During the follow-up, relapse occurred in 69 (85.2%) PSC patients and 220 (65.3%) LUAD patients, and ultimately, 54 (66.7%) and 202 (59.9%) patients died, respectively. There were significantly disparities were noted in PFS (before PSM: P < 0.0001; After PSM: P < 0.0001) and OS (before PSM: P = 0.022; After PSM: P < 0.0001) between patients with PSC and those with LUAD (Fig. 2A, B). Compared to LUAD, PSC has a markedly poorer prognosis.

28 patients were classified as PC and 20 were SCC. There were significantly differences in PFS (P = 0.00036) and OS (P = 0.0053) between PC and SCC (Fig. 2C). Compared to PC, SCC has a poorer prognosis.

**Fig. 3:**
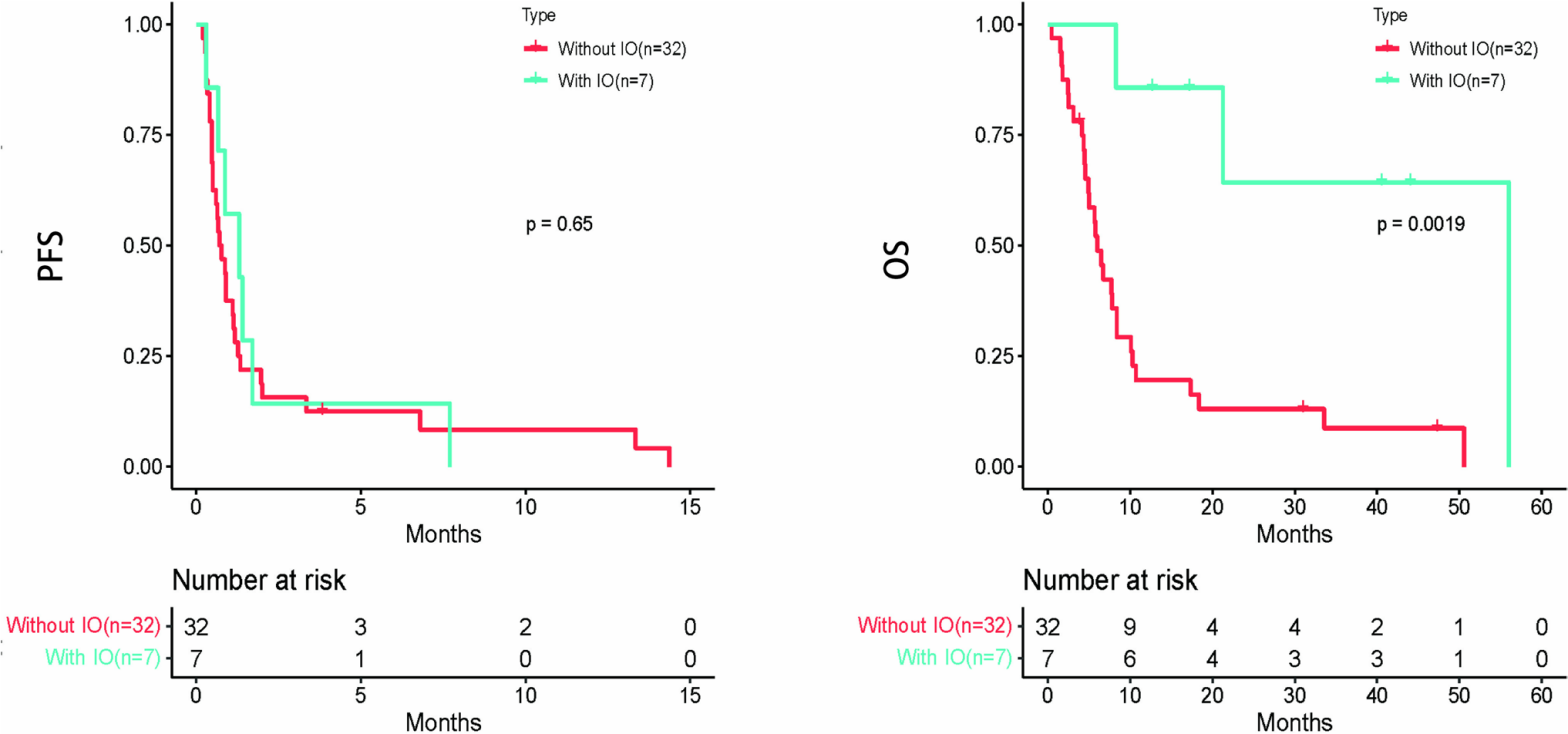
Kaplan–Meier survival curves for progression-free survival (PFS) and overall survival (OS) according to patient treated with Immuno-Oncology (IO) and without IO

42 patients were diagnosed with non-advanced PSC (Stage I-IIIA), among whom 34 underwent surgical treatment, while 8 received chemotherapy only. The Therapeutic schemes were shown in Supplementary Table S5. No differences were observed in either PFS or OS (Fig. S2). 39 of 81 patients were diagnosed as advanced PSC (stage IIIB-IV). 7 of them treated with Immune-Oncology (IO) and 32 of them without IO. 7 patients who received IO, meeting the indications for immunotherapy, had PD-L1 expression levels ≥ 1% [17], with 5 patients treated with pembrolizumab and 2 patients treated with sintilimab. The other 6 patients with PD-L1 expression ≥ 1% did not receive immunotherapy for economic reasons. The clinical characteristics of them were showed in Supplementary Table S6. There were significantly differences in OS (P = 0.0019) for PSC treated with IO compared with those treated without IO (Fig. 3). Immunotherapy significantly extended the overall survival, which brings long-term survival to those with advanced PSC.

## Discussion

Although many authors have reported case studies and clinical feature analyses of PSC, larger sample retrospective studies remain scarce due to the rarity of PSC [18, 19]. The therapeutic approaches outlined in these studies exhibit considerable variation. This study included 111 PSC patients from three academic centers, described the clinical and pathological characteristics of PSC, and compared the efficacy of surgery, chemotherapy, and immunotherapy in patients at different stages. Overall, the majority of patients were male, with a median survival time of 59.6 years. KRAS mutations and EGFR mutations were the two most common mutation types. In advanced PSC patients, chemotherapy combined with immunotherapy led to an improvement in OS compared to chemotherapy alone.

Several previous studies have described the clinical characteristics of PSC patients. Asad Ullah et al. [20] and Susann Stephan-Falkenau et al. [21] have shown a higher proportion of males among PSC patients, with a male-to-female ratio of 1.45:1 and 1.4:1. A study analyzing clinical information of PSC patients from the SEER database from 2000 to 2018 showed that 32.3% of the patients were between 70 and 79 years old [20]. Pleomorphic carcinoma tends to occur more commonly in the right lung, often presenting as peripheral tumors with chest wall invasion. An endobronchial location is uncommon.

PSC showed significant differences from LUAD in terms of PFS and OS. This histological pattern served as a substantial predictor of survival in both univariable analysis and multivariable analysis, which are more aggressive compared to other subtypes of NSCLC [15]. In comparison to LUAD, PSC exhibited significantly poorer PFS (P < 0.0001) and OS (P = 0.022). This result aligns with the findings of Chiara Baldovini et al., who reported that PSC has a lower survival rate compared to adenocarcinoma and squamous cell carcinoma [22]. The previous study regarded PSC as a malignancy associated with an unfavorable prognosis [23]. For patients with PC, those who received surgical resection exhibited a median survival time of 23.6 months, whereas those who did not have 14.9 months [15]. Among PSC patients receiving first-line immunotherapy, the median PFS ranged from 5.0 to 7.2 months, with the median OS ranging from 13.0 to 22.2 months [24, 25]. The 5-year survival rate is 15% [26].

Pleomorphic carcinoma consists of more than 10% spindle and/or giant cells, combined with elements of adenocarcinoma, SCC, or LCC [27, 28]. The PFS (P = 0.00036) and OS (P = 0.0053) of PC were notably superior compared to SCC. Thus, it can be inferred that patients with an increased prevalence of spindle cells experience a worse prognosis, aligning with the findings of Wang et al. [4]. Given the unfavorable prognosis associated with PSC, it is imperative to develop a comprehensive treatment approach. The management of PSC, a malignant neoplasm, still maintains controversial [7, 19, 29, 30]. A study indicated that treatment with surgery was associated with improved survival outcomes [20]. Another study demonstrated that adjuvant chemotherapy was significantly associated with better survival [15]. A multicenter retrospective study analyzed the immunotherapy outcomes of 21 PSC patients and found that first-line immunotherapy shows promising therapeutic potential for PSC treatment [24]. Additionally, a recent study showed that the combination of ICIs with anti-angiogenic agents notably improved PFS [8]. A study based on 179 PSC patients indicated that immunotherapy could prolong survival in PSC patients without gene mutations [21]. A phase II clinical study showed that Savolitinib exhibited encouraging efficacy and a tolerable safety profile in PSC patients with METex14 skipping mutations [7]. In a separate investigation, KRAS mutations as well as EGFR mutations were identified in PSC [31]. The efficacy of KRAS inhibitors or EGFR tyrosine kinase inhibitors in treating these unique NSCLC cases remains uncertain and warrants additional investigation [29]. According to COSMIC data, the most frequently detected mutations were TP53 (31%), ARID1A (23%), and NF1 (17%). A better prognosis was associated with the elevated expression of genes related to TNFα signaling and glycolysis [8].

PSC exhibits both carcinomatous and sarcomatous pathological features. Based on histological types, it is classified into PC, SCC, CS, GCC, and PB. Distinguishing PSC from other types of NSCLC, as well as differentiating among its five subtypes, is a challenging work [15]. We found that the sarcomatoid component often shows positive expression for Vimentin, while the epithelial components exhibit varying degrees of positive expression for CK, TTF-1, CK7, P40, P63, and EMA. A study revealed that in tumors consisting entirely of spindle and/or giant cells, CK7 and TTF-1were positive in 70% and 55% of cases, respectively. In PC containing an epithelial component, the sarcomatoid component exhibited positivity for CK7, TTF-1, and surfactant protein-A in 62.7%, 43.1%, and 5.9% of cases, respectively [27]. Additionally, a study suggested that epithelial-mesenchymal transition (EMT) is crucial in the carcinogenesis of PSC [29]. Additional studies are required to investigate the underlying mechanisms moving forward.

This study has several limitations. Firstly, the sample size of PSC patients in this study is relatively small, and future larger-scale studies are needed to validate and expand our findings. Additionally, although we examined the mutational status of PSC and reported the findings based on a certain number, the sample size remained relatively small. Secondly, the presence of controversial treatment strategies necessitated varying approaches for patients, potentially resulting in divergent outcomes. Thirdly, the median follow-up time in this study is relatively short, and there is an absence of long-term follow-up data. Besides, additional biomarkers or molecular features could not be further explored due to the retrospective nature of the study; however, we will continue to investigate them in prospective research. This might introduce a potential bias.

To summarize, this study revealed that PSC, a malignancy with a low incidence, typically presents with a dismal prognosis and exhibits a higher frequency of KRAS and EGFR mutations. Spindle cell carcinoma has a poorer prognosis. Immunotherapy can extend the survival of patients with advanced PSC.

## Supplementary Information

Below is the link to the electronic supplementary material.

Supplementary file1 (DOCX 315 kb)

## Data Availability

Study data made available upon reasonable request.
