# Supplementary material for: Clinical characteristics and survival outcomes in patients with pulmonary sarcomatoid carcinoma: a multicenter retrospective study
Source: Clin Transl Oncol. 2024 Dec 25;27(7):2993–3000. doi: 10.1007/s12094-024-03823-8 (PMC12178996; doi:10.1007/s12094-024-03823-8)
Supplement: Supplementary file 1 — Supplementary file1 (DOCX 315 kb) [file 12094_2024_3823_MOESM1_ESM.docx]

Supplementary data

The following are the Supplementary data to this article:


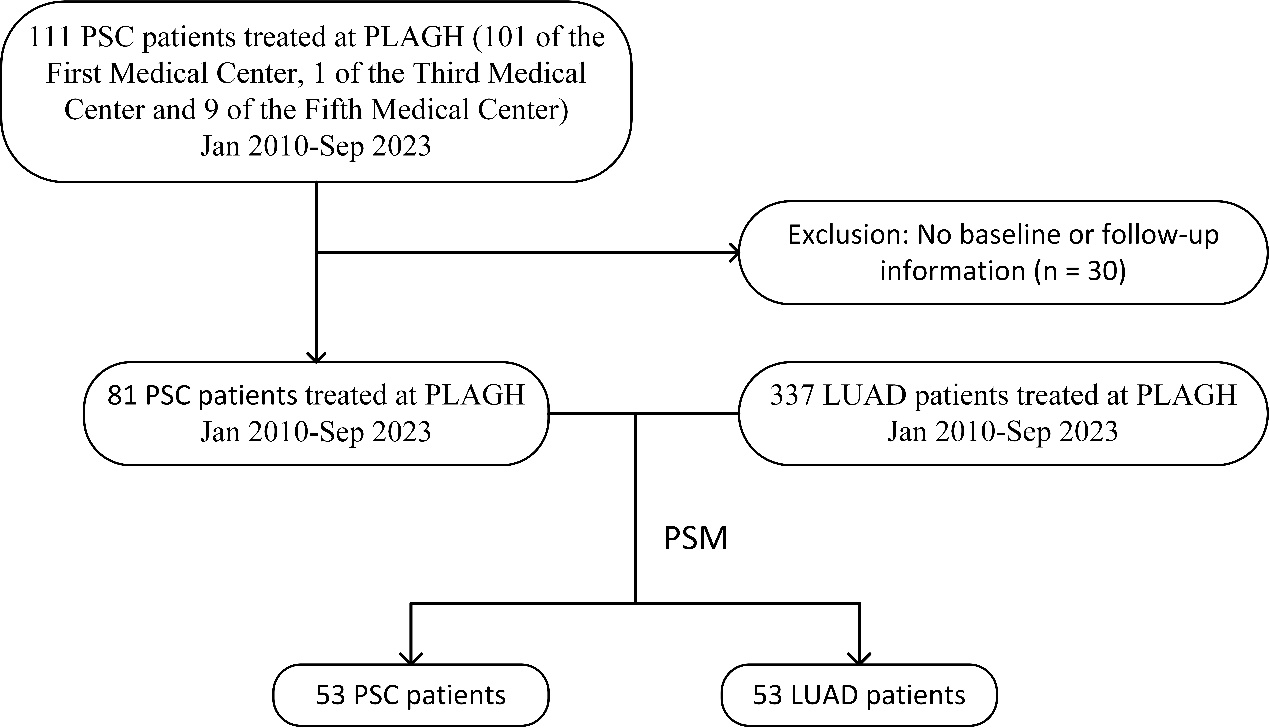


**Figure S1.** **Study flowchart.** LUAD, lung adenocarcinomas; PSC, Pulmonary Sarcomatoid Carcinoma; PSM, Propensity score matching.


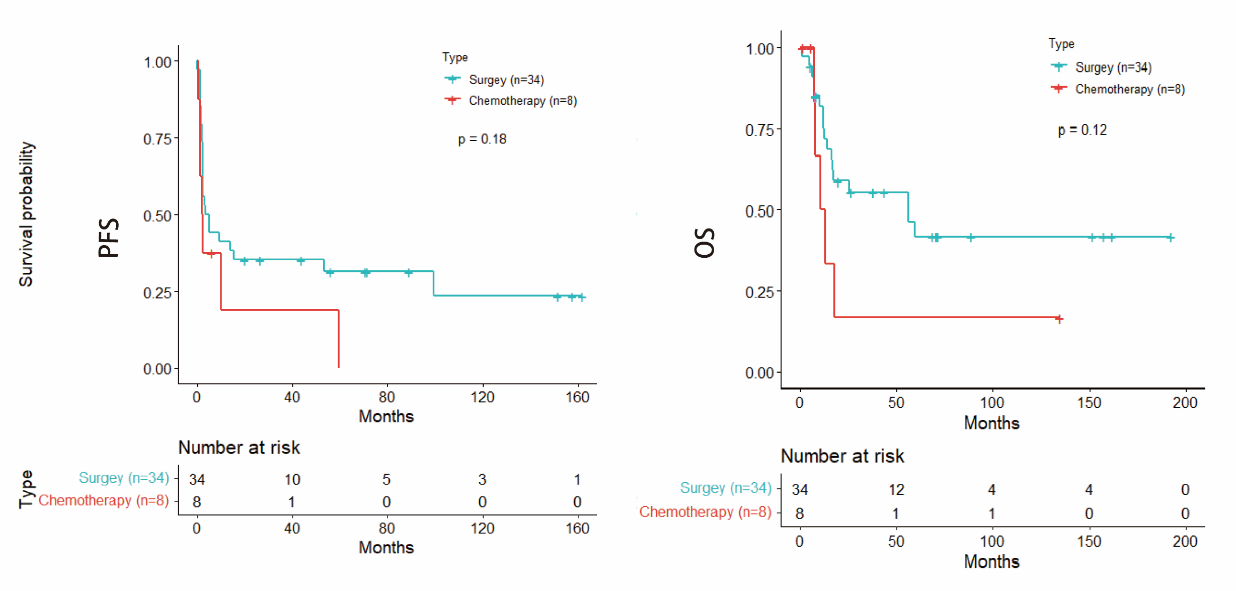


**Figure S2. Kaplan-Meier survival curves** for progression-free survival (PFS) and overall survival (OS) according to non-advanced PSC patients (stage I-IIIA) treated with surgery and chemotherapy.

**Table S1:** Mutational signatures of 19 PSC

| Cases | Age/Sex | Smoking | Stage | Mutataions | Subtype |
| --- | --- | --- | --- | --- | --- |
| 1 | 59/M | Never | IVa | KRAS (G13D) | PSC |
| 2 | 68/M | Smoker | IIb | KRAS (G12D) | PSC |
| 3 | 53/M | Smoker | IVb | Not detected | PSC |
| 4 | 69/M | Smoker | IVa | NF2 | SCC |
| 5 | 72/M | Smoker | IVa | BRAF V600E | PSC |
| 6 | 46/M | Never | IVa | EGFR 19del | PSC |
| 7 | 52/M | Smoker | IVb | SMARCA4 | SCC |
| 8 | 65/M | Never | IIIb | Not detected | GCC |
| 9 | 48/F | Never | IIIa | Not detected | SCC |
| 10 | 57/M | Smoker | IIIa | EGFR 19del | GCC |
| 11 | 61/M | Smoker | IIa | MET amp | CS |
| 12 | 55/M | Smoker | IIb | KRAS (G12C) | PC |
| 13 | 65/F | Never | IVb | KRAS (Q61H) | PC |
| 14 | 64/M | Smoker | IIIa | ERBB4 exon15,  KRAS exon2,  TP53 | PSC |
| 15 | 45/M | Smoker | IIb | EGFR 19del | PSC |
| 16 | 62/M | Smoker | IIIa | Not detected | PC |
| 17 | 44/M | Smoker | IIa | KRAS Exon2 | PC |
| 18 | 73/M | Smoker | IVa | Not detected | PC |
| 19 | 84/M | Smoker | IVa | Not detected | PSC |

Abbreviations: CS, Carcinosarcoma; F, female; GCC, Giant cell carcinoma; M, male; PC, Pleomorphic carcinoma; PSC, Pulmonary sarcomatoid carcinoma; SCC, Spindle cell carcinoma.

**Table S2:** Immunohistochemical staining

| Cases | Positive | Negative | Total | % (positive/total) |
| --- | --- | --- | --- | --- |
| CK | 75 | 5 | 80 | 93.8 |
| Vimentin | 67 | 8 | 75 | 89.3 |
| TTF-1 | 33 | 47 | 80 | 41.3 |
| CK7 | 40 | 20 | 60 | 66.7 |
| P40 | 5 | 25 | 30 | 16.7 |
| P63 | 17 | 39 | 56 | 30.4 |
| EMA | 6 | 6 | 12 | 50.0 |
| PD-L1 | 13 | 10 | 23 | 56.5 |
| Ki-67 | 69 | 6 | 75 | 92.0 |

**Table S3:** Univariable analyses for PFS and OS in patients with PSC and LUAD before PSM

| Variables | PFS | | | OS | | |
| --- | --- | --- | --- | --- | --- | --- |
|  | HR | 95%CI | *P*-Value | HR | 95%CI | *P*-Value |
| Age | 0.980 | 0.777-1.237 | 0.886 | 1.051 | 0.822-1.343 | 0.693 |
| Gender (Male/Female) | 0.937 | 0.724-1.212 | 0.619 | 0.927 | 0.706-1.218 | 0.586 |
| Smoking history | 1.121 | 0.884-1.421 | 0.346 | 1.147 | 0.893-1.474 | 0.284 |
| Tumor family history | 0.994 | 0.749-1.191 | 0.629 | 0.900 | 0.704-1.151 | 0.402 |
| Type | 2.240 | 1.698-2.955 | **<0.001** | 1.422 | 1.050-1.925 | **0.023** |
| cTNM stage | 0.718 | 0.510-1.009 | 0.057 | 0.467 | 0.308-0.708 | **<0.001** |

Abbreviations: HR, hazard ratio; LUAD, lung adenocarcinomas; OS, overall survival; PFS, progress-free survival; PSC, Pulmonary Sarcomatoid Carcinoma; PSM, Propensity score matching.

**Table S4:** Multivariable analyses for PFS and OS in patients with PSC and LUAD before PSM

| Variables | PFS | | | OS | | |
| --- | --- | --- | --- | --- | --- | --- |
|  | HR | 95%CI | *P*-Value | HR | 95%CI | *P*-Value |
| Age | 0.945 | 0.743-1.202 | 0.647 | 0.987 | 0.764-1.275 | 0.921 |
| Gender (Male/Female) | 0.934 | 0.697-1.252 | 0.648 | 0.977 | 0.715-1.335 | 0.883 |
| Smoking history | 1.061 | 0.808-1.392 | 0.672 | 1.184 | 0.888-1.579 | 0.249 |
| Tumor family history | 0.862 | 0.679-1.094 | 0.221 | 0.837 | 0.650-1.077 | 0.166 |
| Type | 7.273 | 5.002-10.535 | **<0.001** | 2.850 | 2.005-4.052 | **<0.001** |
| cTNM stage | 0.181 | 0.115-0.287 | **<0.001** | 0.260 | 0.162-0.416 | **<0.001** |

Abbreviations: HR, hazard ratio; LUAD, lung adenocarcinomas; OS, overall survival; PFS, progress-free survival; PSC, Pulmonary Sarcomatoid Carcinoma; PSM, Propensity score matching.

**Table S5:** Therapeutic schemes of 81 PSC patients

|  | Stage I-IIIA (n=42) | Stage IIIB-IV (n=39) |
| --- | --- | --- |
| Surgery | 23 (54.8) | 0 |
| Surgery combined with adjuvant chemotherapy | 11 (26.2) | 0 |
| Chemotherapy | 8 (19.0) | 32 (82.1) |
| Chemotherapy combined with Immunotherapy | 0 | 7 (17.9) |

Abbreviations: PSC: Pulmonary Sarcomatoid Carcinoma.

**Table S6:** Clinical characteristics of patients treated with IO and without IO

|  | With IO（n=7） | Without IO（n=32） | P Value |
| --- | --- | --- | --- |
| Age |  |  | 0.525 |
| < 60 | 4(22.2) | 14(77.8) |  |
| ≥60 | 3(14.3) | 18(85.7) |  |
| Sex |  |  | 0.292 |
| Male | 5(15.2) | 28(84.8) |  |
| Female | 2(33.3) | 4(66.7) |  |
| Smoking history |  |  |  |
| Ever | 4(14.8) | 23(85.2) | 0.450 |
| Never | 3(25.0) | 9(75.0) |  |
| Tumor family history |  |  | 0.981 |
| Yes | 5(17.9) | 23(82.1) |  |
| No | 2(18.2) | 9(81.8) |  |
| Gene Mutation |  |  | 0.174 |
| Yes | 2(40.0) | 3(60.0) |  |
| No | 5(14.7) | 29(85.3) |  |
| cTNM stage |  |  | 0.503 |
| I-IIIA | 0(00.0) | 2(100.0) |  |
| IIIB-IV | 7(18.9) | 30(81.1) |  |

Abbreviations: LUAD, lung adenocarcinomas; PSC: Pulmonary Sarcomatoid Carcinoma; IO: Immuno-Oncology.
